# Supplementary material for: Targeted metabolomics suggests a probable role of the FTO gene in the kynurenine pathway in prediabetes
Source: PeerJ. 2022 Jun 21;10:e13612. doi: 10.7717/peerj.13612 (PMC9231341; doi:10.7717/peerj.13612)
Supplement: Supplemental Information 2 [file peerj-10-13612-s002.docx]

| Heading | Meaning |
| --- | --- |
| Gender | 1 = Male, 2 = Female |
| Fto_1  (FTO rs9939609 A) | 1 = present, 0 = absent |
| Fto_2  (FTO rs9939609 T) | 1 = present, 0 = absent |
| FTO  (FTO genotype) | 1 = AA, 2 = AT, 3 = TT |
